# Similarities in butterfly emergence dates among populations suggest local adaptation to climate

**DOI:** 10.1111/gcb.12920

**Published:** 2015-06-17

**Authors:** David B. Roy, Tom H. Oliver, Marc S. Botham, Bjorn Beckmann, Tom Brereton, Roger L. H. Dennis, Colin Harrower, Albert B. Phillimore, Jeremy A. Thomas

**Affiliations:** ^1^ Centre for Ecology & HydrologyWallingfordOxfordshireOX10 8BBUK; ^2^ Butterfly ConservationManor YardEast LulworthWarehamDorsetBH20 5QPUK; ^3^ Institute for Environment, Sustainability and RegenerationStaffordshire UniversityRoom s122Mellor BuildingCollege RoadStoke on TrentST4 2DEUK; ^4^ Institute of Evolutionary BiologyThe King's BuildingsEdinburghEH9 3JTUK; ^5^ Department of ZoologyUniversity of OxfordSouth Parks RoadOxfordOX1 3PSUK

**Keywords:** butterfly monitoring scheme, climate change, life history, local adaptation, phenology, plasticity, temperature, traits

## Abstract

Phenology shifts are the most widely cited examples of the biological impact of climate change, yet there are few assessments of potential effects on the fitness of individual organisms or the persistence of populations. Despite extensive evidence of climate‐driven advances in phenological events over recent decades, comparable patterns across species' geographic ranges have seldom been described. Even fewer studies have quantified concurrent spatial gradients and temporal trends between phenology and climate. Here we analyse a large data set (~129 000 phenology measures) over 37 years across the UK to provide the first phylogenetic comparative analysis of the relative roles of plasticity and local adaptation in generating spatial and temporal patterns in butterfly mean flight dates. Although populations of all species exhibit a plastic response to temperature, with adult emergence dates earlier in warmer years by an average of 6.4 days per °C, among‐population differences are significantly lower on average, at 4.3 days per °C. Emergence dates of most species are more synchronised over their geographic range than is predicted by their relationship between mean flight date and temperature over time, suggesting local adaptation. Biological traits of species only weakly explained the variation in differences between space‐temperature and time‐temperature phenological responses, suggesting that multiple mechanisms may operate to maintain local adaptation. As niche models assume constant relationships between occurrence and environmental conditions across a species' entire range, an important implication of the temperature‐mediated local adaptation detected here is that populations of insects are much more sensitive to future climate changes than current projections suggest.

## Introduction

Evidence is accumulating that climate change is already affecting wildlife across the globe and across ecosystems (Parmesan *et al*., 2013; Settele *et al*., 2014). Phenological responses have been particularly well documented, revealing a general trend that spring events in the northern hemisphere have become earlier for several species groups (Parmesan, 2007). Such changes have the potential to disrupt the synchrony of ecological interactions (Thackeray *et al*., 2010) or lead to maladaptive changes with implications for population persistence (Van Dyck *et al*., 2015).

Populations can persist under a changing environment if they have dispersal capacity to track a shifting optimum through space, or can persist *in situ* by evolving to the new local conditions, or possess sufficient phenotypic plasticity to track a shifting optima (Chevin *et al*., 2010). The combination of rapid climate change and habitat fragmentation due to human activity may prevent many species from tracking the climate to which they are currently adapted through dispersal (Jump & Peñuelas, 2005). The evolutionary potential of populations and relative contribution of local adaptation and phenotypic plasticity to geographic variation are therefore key factors in understanding the limits to population persistence (Chevin *et al*., 2010). For instance, populations that differ in phenology due to temperature‐driven local adaptation are expected to be subject to directional selection if the climate changes and population persistence will depend on the degree to which absolute fitness is reduced and the capacity of the population for adaptive evolution. In comparison, if populations are able to track the optimum via plasticity, mean population fitness may not be affected (Phillimore *et al*., 2010).

Evidence for local adaptation among populations has traditionally been derived from labour intensive and logistically challenging reciprocal transplant experiments and is only available for a taxonomically biased handful of species (Hereford, 2009). The application of recently developed statistical techniques that decompose spatiotemporal phenological data into contributions of phenotypic plasticity and local adaptation with respect to an environmental gradient provides a relatively straightforward alternative (Phillimore *et al*., 2010). Applying this approach to monitoring data allows local adaptation to be estimated for a suite of species with differing life‐history characteristics.

Standardised monitoring of butterflies has operated in the UK for over three decades and has revealed temperature‐related changes in abundance (Roy *et al*., 2001) and population dynamics (Oliver *et al*., 2012). Changes in flight dates of UK butterflies have been remarkably consistent, with almost all species showing a marked advance in the timing of adult emergence with increasing temperature (Roy & Sparks, 2000). To date, there have been few multi‐species analyses of spatial variation in insect phenology apart from the demonstration that aphid flight times (Zhou *et al*., 1995) and butterfly sighting dates (Roy & Asher, 2003) are related to geographic gradients in temperature. Even fewer studies have assessed spatial and temporal trends simultaneously (Kharouba *et al*., 2013), yet this is key to accurate estimates of the survival of species, either within a region or globally, under future climates (Hodgson *et al*., 2011). For if the observed relationship between phenology and temperature in a species is closely matched over space and time (consistent with phenotypic plasticity determining both patterns), it is reasonable to assume that the development rates and fitness of individuals will respond to climate warming in similar and predictable ways throughout its current and potential geographical ranges. In contrast, if a species contains subsets of genotypes, each adapted to function optimally under different local climates any future responses will be harder to predict (Visser, 2008) and selection may impact negatively on demography (Chevin *et al*., 2010).

Here, we present the first test of local adaptation for a whole faunal group within a region. We use the largest and longest‐running data set on insect populations, the UK's Butterfly Monitoring Scheme (UKBMS), to quantify changes in butterfly flight dates over a 37 year period (1976–2012) at 1622 sites. We test the hypothesis that within‐ and among‐population slopes between mean flight dates and temperature are equivalent, implying that any geographic covariation is solely due to plasticity. We analyse differences in trends in phenology–temperature relationships in relation to species' ecological traits in order to infer potential mechanisms that explain local adaptation.

## Materials and methods

### Data sources

Daily counts of butterflies were obtained from the UKBMS. The methodology of this scheme is described in detail by Pollard & Yates (1993) and is summarised only briefly here. At each site, ideally a fixed route is walked in each of 26 recording weeks from 1 April to 29 September, provided weather conditions meet set criteria and volunteers are able to do a transect walk. All butterflies seen within fixed limits are recorded. The raw data used for this study are counts and days of counts, numbered from 1 April for the period 1976–2012. Data were available from 1622 sites distributed across the UK (Fig. S1). Average monthly temperatures from 1975 to 2012 for 5‐km^2^ grid cells of the British Ordnance Survey national grid were obtained from the UK Climate Projections 2009 data set (http://ukclimateprojections.metoffice.gov.uk/).

### Calculation of phenology measures

The timing of each flight period was measured as the (weighted) mean date of counts, as described by Brakefield (1987), and gives an estimation of the date of mean abundance in the adult flight period (van Strien *et al*., 2008). The day of the butterfly counts was used as the unit of time, providing a more precise measure of phenology than previous analyses of butterfly transect schemes, which used recording weeks (e.g. Roy & Sparks, 2000). We restrict our analyses to site‐year‐species combinations where there is sufficient data to estimate an annual index abundance for a flight period (Rothery & Roy, 2001) and therefore provide a robust measure of mean flight dates.

Most butterfly species in the UK have a single generation per year, allowing phenological measures to be derived from a distinct flight period. Two univoltine species, *Aglais io* and *Gonepteryx rhamni*, overwinter as adults. Individuals of these species appear throughout the winter months, but mainly in March–April, partly before the monitoring season begins. The subsequent single generation emerges in the summer months, and we restrict analyses to this prehibernation period for these two species.

A number of species show a more complex pattern of adult emergences. We excluded multivoltine species with a flight period that is characterised by two or more overlapping generations that can not readily be separated (e.g. *Pararge aegeria*,* Coenonympha pamphilus*,* Aglais urticae*,* Leptidea sinapis*,* Polygonia c‐album*). Several other multivoltine species have a distinct first generation in the spring that is followed by one or more, often overlapping, generations throughout the summer and early autumn. For most species, this makes it impossible to identify distinct generations for second and subsequent generations, so phenology measures were calculated for the spring generation only. Finally, we excluded species whose populations in the UK mainly comprise migrant individuals (e.g. *Vanessa atalanta*,* Vanessa cardui*,* Colias croceus*). In total, 31 species were analysed.

### Statistical analysis

#### Identifying the most important temperature period for each species' phenology

For each UKBMS monitoring site, monthly temperatures were obtained from the 5 km grid cell containing the site centroid. Three‐monthly running means were then calculated by taking a mean of the temperature of each month and its preceding and following month. We used these running means to identify for each species the three‐month period whose mean temperature had the greatest effect on flight date UK‐wide. To do this, we fitted 12 separate regression models with the UK‐wide mean flight date in each year as the response variable and mean temperature from one of the 12 three‐monthly periods as an explanatory variable. We included periods that come after the flight period of the species to test that spurious relationships were not apparent. We selected the model with the strongest relationship (as measured by t‐value) between temperature and mean flight date (Table 1). The same or adjacent month was selected using the magnitude of the effect (coefficient between temperature and mean flight date) as an alternative criterion (Table S1) and gave similar results (Fig. S2). For example, for *Callophrys rubi* period 3 (March) had the largest, most statistically significant (negative) coefficient, indicating that warmer mean monthly temperatures between February and April had the greatest effect on shifting the butterfly mean flight date to earlier in the year. The t‐values and coefficients of the relationships between mean flight dates and all 12 three‐monthly periods tested are given in the Supplementary Materials (Table S1). We adopt this data‐driven approach to negate incomplete knowledge of the biology of butterfly species; the detailed autecological information needed to identify key stages of development is only available for a few species.

**Table 1:** Trends over time (within‐population slope) and space (among‐population slope) in the relationship between mean flight date and temperature. Shifts in mean flight dates are number of days per 1 °C change in temperature and are modal means from a Bayesian mixed model (see methods section), with 95% confidence intervals in brackets. Slope estimates are emboldened where the 95% confidence interval does not span zero. The overall mean flight date and the three‐monthly period with the strongest relationship between flight date and temperature are also given for each species

Species	Mean flight date	Month	*n* (sites)	*n* (site:year)	*R* ^2^	Within‐population slope	Among‐population slope	Slope difference
Group (a): Univoltine species: one flight period in each year								
*Thymelicus sylvestris*	21th June	May	1033	6121	0.25	−**6.41 (**−**8.29;** −**4.74)**	−**4.06 (**−**5.10;** −**3.17)**	**2.38 (0.35; 4.21)**
*Hesperia comma*	18th July	July	58	553	0.21	−**4.35 (**−**5.75;** −**3.14)**	2.26 (−5.88; 10.79)	6.53 (−1.62; 15.46)
*Ochlodes sylvanus*	5th June	May	1140	8126	0.16	−**6.76 (**−**8.18;** −**5.52)**	−0.15 (−1.90; 1.30)	**6.63 (4.70; 8.56)**
*Erynnis tages*	26th April	March	449	2785	0.26	−**5.68 (**−**6.71;** −**4.70)**	−1.79 (−3.56; 0.02)	**3.88 (1.86; 5.87)**
*Pyrgus malvae*	22nd April	March	344	2041	0.10	−**6.00 (**−**7.11;** −**4.57)**	−1.44 (−13.82; 9.68)	4.51 (−7.70; 16.14)
*Anthocharis cardamines*	10th April	March	1209	7422	0.39	−**7.21 (**−**8.35;** −**6.09)**	−5.23 (−6.75; −3.78)	**2.04 (0.24; 3.92)**
*Callophrys rubi*	23rd April	March	514	2784	0.19	−**5.42 (**−**6.11;** −**4.59)**	**3.66 (1.31; 6.43)**	**9.12 (6.42; 11.85)**
*Polyommatus coridon*	10th July	June	233	1765	0.18	−**5.22 (**−**6.53;** −**3.78)**	−2.67 (−6.75; 1.01)	2.47 (−1.36; 6.97)
*Limenitis camilla*	15th June	May	278	1745	0.21	−**7.83 (**−**8.60;** −**6.86)**	−12.68 (−25.73; 5.73)	−4.85 (−19.12; 12.4)
*Boloria selene*	21th May	May	227	1228	0.29	−**6.25 (**−**8.19;** −**4.30)**	−**6.26 (**−**8.11;** −**4.08)**	−0.03 (−2.63; 2.71)
*Boloria euphrosyne*	26th April	March	157	872	0.23	−**6.96 (**−**8.15;** −**5.71)**	−**5.02 (**−**9.11;** −**0.74)**	1.9 (−2.23; 6.24)
*Argynnis adippe*	17th June	May	62	478	0.12	−**6.22 (**−**8.51;** −**4.69)**	−7.07 (−19.11; 4.66)	−0.89 (−12.39; 11.59)
*Argynnis aglaja*	19th June	May	447	2312	0.09	−**4.62 (**−**6.08;** −**3.04)**	−**1.79 (**−**3.22;** −**0.32)**	**2.77 (0.61; 4.86)**
*Argynnis paphia*	26th June	May	519	2670	0.19	−**5.53 (**−**7.21;** −**3.84)**	−**7.53 (**−**10.18;** −**4.88)**	−2.01 (−5.19; 0.94)
*Euphydryas aurinia*	3rd May	April	87	474	0.38	−**6.97 (**−**9.35;** −**5.15)**	−**8.24 (**−**13.55;** −**2.69)**	−1.26 (−7.05; 4.78)
*Melanargia galathea*	13th June	May	721	4866	0.37	−**6.92 (**−**8.13;** −**5.78)**	−**4.72 (**−**7.38;** −**2.33)**	2.21 (−0.60; 4.87)
*Hipparchia semele*	6th July	May	231	1310	0.05	−**3.74 (**−**5.31;** −**2.37)**	1.20 (−1.40; 3.54)	**5.04 (2.43; 7.93)**
*Pyronia tithonus*	2nd July	June	1142	8391	0.45	−**6.13 (**−**6.96;** −**5.33)**	−**3.39 (**−**4.41;** −**2.37)**	**2.77 (1.46; 4.05)**
*Maniola jurtina*	23th June	May	1361	9876	0.13	−**4.54 (**−**5.93;** −**3.01)**	−**1.04 (**−**1.89;** −**0.10)**	**3.51 (1.84; 5.28)**
*Aphantopus hyperantus*	13th June	May	1202	7546	0.37	−**6.52 (**−**7.38;** −**5.64)**	−**2.02 (**−**2.79;** −**1.42)**	**4.51 (3.42; 5.58)**
Group (b): Univoltine species: two flight periods per year but only one generation (adults overwintering, summer generation analysed)								
*Gonepteryx rhamni*	11th July	July	877	5720	0.30	−**8.31 (**−**9.95;** −**6.77)**	−**5.72 (**−**7.78;** −**3.45)**	2.65 (−0.17; 5.27)
*Aglais io*	8th July	May	1253	8273	0.53	−**7.60 (**−**9.71;** −**5.68)**	−**8.82 (**−**9.66;** −**7.9)**	−1.18 (−3.38; 0.82)
Group (c): Multivoltine species: two or more flight periods per year representing different generations (1st generation analysed)								
*Pieris rapae*	20th April	April	1190	7325	0.31	−**9.07 (**−**10.74;** −**7.32)**	−**3.72 (**−**4.71;** −**2.8)**	**5.38 (3.35; 7.37)**
*Pieris napi*	18th April	April	1214	7986	0.41	−**8.21 (**−**9.78;** −**6.90)**	−**5.18 (**−**5.89;** −**4.61)**	**3.01 (1.41; 4.54)**
*Pieris brassicae*	25th April	April	1195	7239	0.29	−**8.10 (**−**9.51;** −**6.91)**	−**5.45 (**−**6.39;** −**4.34)**	**2.67 (0.84; 4.23)**
*Lycaena phlaeas*	25th April	March	887	3739	0.31	−**6.73 (**−**7.75;** −**5.65)**	−**4.35 (**−**5.61;** −**2.99)**	**2.40 (0.78; 4.06)**
*Cupido minimus*	8th May	March	169	938	0.28	−**4.98 (**−**6.32;** −**3.70)**	−**4.65 (**−**7.14;** −**2.07)**	0.32 (−2.6; 3.04)
*Aricia agestis*	1st May	March	411	2005	0.30	−**6.23 (**−**7.30;** −**5.09)**	−**5.68 (**−**8.44;** −**3.08)**	0.56 (−2.15; 3.6)
*Polyommatus icarus*	10th June	April	1104	6529	0.55	−**7.04 (**−**8.52;** −**5.21)**	−**12.92 (**−**14.55;** −**11.2)**	−5.90 (−8.25; −3.66)
*Celastrina argiolus*	12th May	March	908	4246	0.18	−**5.89 (**−**6.86;** −**4.83)**	−**3.62 (**−**6.43;** −**0.54)**	2.17 (−0.91; 5.36)
*Lasiommata megera*	27th May	March	420	1981	0.39	−**7.33 (**−**8.09;** −**6.57)**	−**6.14 (**−**7.71;** −**4.57)**	1.18 (−0.54; 2.88)

#### Comparing effects of temperature change on species' phenology over space and time

To compare the relative effect of temperature change over time vs. temperature change over space, we followed the method of Phillimore *et al*. (2010). Local monitoring sites were aggregated into ‘populations’ by overlaying a 50 km grid (N‐S/E‐W orientation) onto the UK map. We also repeated the analysis at two further scales, with similar results: using individual site location (e.g. no aggregation) and by overlaying with a 100 km grid, finding the results to be highly correlated between scales (Fig. S3).

The three‐monthly mean temperature for each site‐by‐year combination, along with respective mean flight date, were fitted as response variables in a bivariate Bayesian mixed model framework (MCMCglmm; Hadfield, 2010), with population, year and residual fitted as random effects. The model was run for 13 000 iterations with a burn‐in of 3000 iterations. Priors for the (co)variance components were weakly informative and followed the inverse‐Wishart distribution with V = 1 and *ν* = 0.002. For each random term, dividing the estimated covariance between temperature and flight date by the variance component for temperature gives an estimate of the slope of the regression of phenology on temperature (Phillimore *et al*., 2010). A slope through time (within population) and space (among population) was estimated from the year and population random effects, respectively.

**Figure 1:**
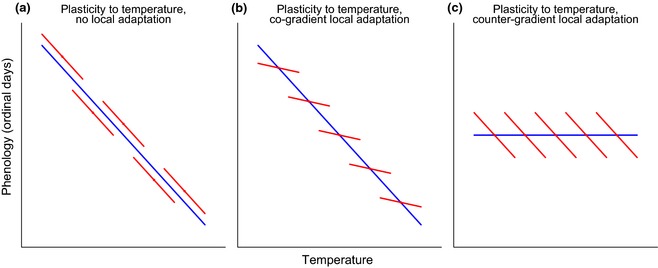
A schematic showing the interpretation of three forms of spatial (blue) and temporal (red) slopes for the population‐level response of phenology to a temperature cue. (a) Temporal and spatial slopes are the same, consistent with the expectation if phenotypic plasticity is responsible for the spatial slope. (b) The spatial slope is steeper than the temporal slope, as expected under co‐gradient local adaptation. (c) The spatial slope is shallower than the temporal slope, as expected under countergradient local adaptation.

Assuming that all populations share the same plastic response of flight date to temperature and that the contribution of microevolution to the within‐population slope has been minimal over the 37 years of the monitoring scheme, then the slope of flight date on temperature over time (‘within‐population slope’) should capture a species' temperature‐mediated plasticity in emergence time (Fig. 1a). In comparison, the among‐population slope should capture temperature‐mediated plasticity plus any effect of adaptation of flight dates to local temperatures. Therefore, the difference (∆*b*) between the within‐population slope and the ‘among‐population slope’ estimates the direction and strength of local adaptation (Fig. 1b, c).

The null hypothesis is that within‐ and among‐population slopes are the same (∆*b ≈ 0*), implying that any geographic covariation between temperature and flight date is solely due to plasticity. The null hypothesis is rejected if the 95% highest posterior density (HPD) of ∆*b* does not include zero. As stated above, a key assumption of the approach we have taken is that plasticity of flight date does not vary markedly among populations. A visual inspection of the variation of within‐population slopes shows that the plastic response to temperature is largely consistent among populations (Fig. S4). In practice, it is likely that differences in slopes may result from differences in site (i.e. slope/aspect, habitat type and quality) and landscape (i.e. configuration and connectivity of habitat parcels) characteristics; such effects merit more study in future but are secondary to the temperature effects we assess here.

#### Developing a phylogeny of British butterflies

In order to carry out a phylogenetic comparative analysis on species' phenological responses to temperature, we used published molecular data to create phylogenies of British butterflies. We used Geneious (Drummond *et al*., 2006) to search GenBank for nucleotide sequences from the mitochondrial cytochrome oxidase subunit I (COI) gene. We were able to find sequences for 54/62 British species; for a further five species, we included the sequence of a congener (see Fig. S5 legend). Sequences ranging from 406 to 1450 bp long were aligned by eye in Se‐Al (http://tree.bio.ed.ac.uk/software/seal/). Technical details for developing the phylogenies of British butterflies are included within the supplementary material.

#### Comparing trends across species

We used a phylogenetic meta‐analysis (Hadfield & Nakagawa, 2010) to estimate the phylogenetic signal in interspecific variation in the within‐population slope (an estimate of plasticity) and ∆*b* (an estimate of local adaptation) and to test for fixed effect predictors of these values. We implemented this using the *MCMCglmm* R library (Hadfield, 2010) fitting phylogeny as a random effect (Eqn (1)).

(1) $$y_{i}=\mu +\beta x_{i}+a_{i}+e_{i}+m_{i}$$

The trait, *y* (estimate of either plasticity or local adaptation), of species *i* is given by the grand mean (*μ*), plus the influence of any fixed effects (*βx*_*i*_), deviations due to phylogeny (*a*_*i*_), species‐specific residual (*e*_*i*_) and measurement error (*m*_*i*_). *a* and *e* are assumed to follow normal distributions, and their variances ($\sigma_{a}^{2}$ and $\sigma_{e}^{2}$) are estimated in the model. The model incorporates uncertainty in our estimates of the species‐specific measurement error variance (i.e. the variance in the relevant posterior distribution) plasticity and local adaptation. The distribution of *m* was given by

(2) m∼N(0,M)

where **M** is a N x N matrix with the measurement error variances on the off‐diagonal. In addition, we incorporated uncertainty in the phylogenetic hypothesis, by estimating all mixed model fixed and random effects from 1000 trees sampled from the posterior distribution. This meant that the resulting posterior distribution incorporated both model and phylogeny uncertainty (Pagel & Lutzoni, 2002). Phylogenetic heritability was estimated as:

$$H^{2}=\sigma_{a}^{2}/\left(\sigma_{a}^{2}+\sigma_{e}^{2}\right).$$

The biological traits we tested as predictors of the within‐population slope and ∆b were as follows: the seral stage of host plant(s) (early/mid/late succession grasses or trees/shrub); larval development duration (days); degree of multivoltinism (single‐brooded, single plus partial second brooded, double‐brooded, multi‐brooded); hibernation stage (egg, larva, pupa, adult); mobility (sum of binary states for nine attributes including ex‐habitat vagrants, garden records, urban central business district records, at‐sea records, mass movements, range expansions, overseas migration from continent to Europe, regular reversed long distance migration, over‐ocean (Atlantic) migration). The seral stage of host plants was categories following Thomas (2007); all other traits were derived from Dennis *et al*. (2004).

## Results

For all 31 butterfly species analysed, annual fluctuations in mean flight date were strongly related to temperature, with advanced timing in warmer years (Table 1; range of −3.7 to −9.1 days °C^−1^). The temperature variable most correlated with mean flight date for every species was mean temperature averaged over a three‐month period prior to the overall average flight date. In all cases, the magnitude and statistical significance of this response was only slightly reduced in preceding and subsequent three‐month periods (Table S1), due to the intercorrelation in temperatures between months.

Most (28 of 31) species also had a negative relationship between mean flight date and temperature over space, with later flight dates in cooler parts of their range (Table 1). Among‐population temperature–phenology slopes ranged from +3.7 days °C^−1^ in *Callophrys rubi* to −12.9 days °C^−1^ in *Polyommatus icarus*.

**Figure 2:**
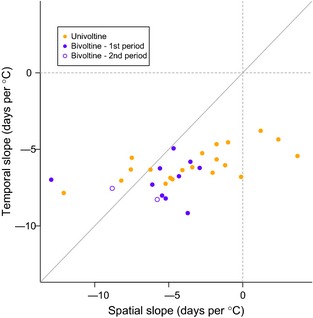
Expected shift in mean flight date for a 1 °C increase in mean temperature in both the spatial (*x*‐axis) and temporal dimension (*y*‐axis) for the 31 species analysed. The line of unity indicates the null hypothesis that temperature change over both space and time has the same effect on phenological shift.

For all species combined, temperature‐related changes in flight periods were greater over time than space (Fig. 2; mean phylogenetically corrected difference = 2.38 (95% CIs: 0.76–3.90) days °C^−1^) and this slope difference was individually significant (95% confidence intervals do not span zero) for 14 species (Table 1). Thus, the emergence dates of most species are more synchronised over their geographic range than is predicted by their relationship between mean flight date and temperature over time (i.e. 26 of 31 species fall below the unity line in Fig. 2). Responses to a 1 °C variation in temperature were almost invariably greater over time than over space. For example, populations of *Ochlodes sylvanus* have appeared on average 1 week earlier per 1 °C increase in May temperatures over the last three decades (within‐population slope). In contrast, populations of this species appear across the country at approximately the same time each year (among‐population slope approximately zero; Table 1).

Within the data set analysed, the absolute temperature range of species is typically higher over space (among‐populations) than over time (within populations), although the interquartile range is more similar and marginally higher over time (Table S2). The mean absolute range values are among‐populations = 6.4 °C vs. within‐populations = 3.5 °C; whereas the mean interquartile ranges are 0.9 °C vs. 1.2 °C, respectively.

**Figure 3:**
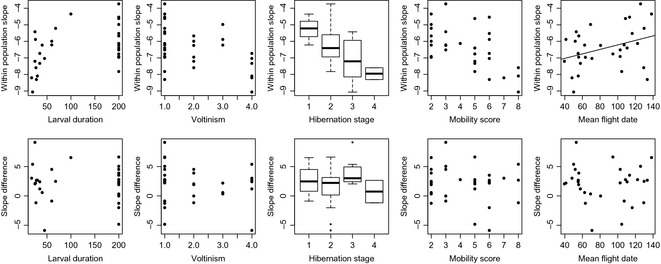
Relationship between butterfly traits and a) species' within‐population phenology–temperature slopes (an estimate of plasticity in flight date; top panels) and slope differences ∆*b* (an estimate of local adaptation; bottom panels). Traits are as follows (panels left to right): larval development duration (days); degree of multivoltinism (single‐brooded, single plus partial second brooded, double‐brooded, multi‐brooded); hibernation stage (egg, larva, pupa, adult); mobility (sum of ranked scores for nine variables); mean flight date (weighted mean of adult abundance by week). Mean flight date was derived directly from monitoring data; all other traits were derived from Dennis *et al*. (2004).

Our analysis of species' traits does not identify a strong link between species' life‐history characteristics and the degree of local adaptation, as measured by the difference in the within‐ and among‐population relationships between appearance dates and temperature (Fig. 3, Table S3). The one significant relationship detected was between the temperature–phenology trend over time (within populations, a measure of the strength of the relationship over time) and the mean timing of appearance, with flight dates of early season species tending to respond more markedly to year‐to‐year temperature differences. We find a significant phylogenetic signal, H^2^ = 0.75 (95% CI: 0.25–0.94) in temperature–phenology relationships over time (within‐population slopes) suggesting that responses are predictable on the basis of relatedness among species. As for the slope difference ∆b, we did not detect a significant phylogenetic signal (h^2^ = 0.06, 95% CI = 0.01–0.90).

## Discussion

We provide the first evidence from a multi‐species analysis of structured monitoring data that geographic relationships between phenology (mean butterfly flight dates) and temperature are not readily predicted from relationships over time. This confirms similar patterns found for sparser, less structured phenology estimates derived from museum specimens (Kharouba *et al*., 2013). The key features of our analyses being data to estimate phenology based on repeated counts from fixed locations over 37 years, with data points per species being three orders of magnitude greater; ~3000 data points per species in our analyses vs. ~2 per species in Kharouba *et al*. (2013). We found that flight periods were earlier in years when the weather was warm prior to and during emergence, confirming the strong relationship previously demonstrated for butterflies (Roy & Sparks, 2000). However, emergence dates of most species are more synchronised over their geographic range than is predicted by their relationship between mean flight date and temperature over time, suggestive of local adaptation to temperature.

The difference between spatial and temporal phenology responses may relate to one or more of the latitudinal gradients reported in butterfly populations, such as in morphology, resource use, life‐history patterns, physiology, biochemistry and behaviour (Nylin, 2009). For example, it is notable that the most extreme exception to the general pattern in Fig. 2, *Callophrys rubi* is the only polyphagous species analysed to switch its principal larval foodplant over its latitudinal range in Great Britain (Thomas, 2007). Having restricted our analyses to a single generation for species with variable voltinism, the other potential mechanisms underpinning local adaptation can broadly be classed into three nonexclusive types: (i) Developmental compensation – for example, pupation can occur at a lower weight, at the cost of producing smaller adults, when temperatures are cool, especially where other cues are in operation such as resource availability or a day‐length trigger for metamorphosis (Dennis & Shreeve, 1989; Van Dyck *et al*., 2015). Complete phenotypic plasticity in development with respect to temperature would lead to similar phenology–temperature relationships across time and space. (ii) Behavioural compensation for cooler temperatures by thermo‐regulating as adults or larvae (Weiss *et al*., 1988), or by occupying warmer or cooler niches, respectively, within ecosystems in regions, seasons or years when air temperatures are lower or higher (Thomas, 1993; Thomas *et al*., 1999, 2001; Roy & Thomas, 2003; Oliver *et al*., 2009, 2012). Note that this latter behavioural mechanism could plausibly arise via the action of another plastic trait. (iii) Physiological or morphological adaptations by local populations to regional climates, whereby each genotype has evolved to function optimally under the range of environmental conditions that it has historically experienced in a region (Nylin & Gotthard, 1998).

If complete phenotypic plasticity and the same reaction norms existed in populations across species' ranges, as postulated by the two explanations involving compensation (developmental and behavioural), then any phenology–temperature relationship should be similar across space and time. For example, not only do many UK butterflies shift to inhabit warmer, narrower niches within sites at higher (cooler) latitudes (Thomas, 1993; Thomas *et al*., 1999, 2001; Roy & Thomas, 2003; Oliver *et al*., 2009), but a similar temporal shift also occurs within individual sites, with females distributing eggs during warm years on foodplants growing in spots that (in our examples) would normally be too cool for exploitation, or compensating for cold weather by concentrating the population into the warmest available microhabitats (Thomas *et al*., 1994, 2001; Roy & Thomas, 2003). Similarly, phenology has been shown to be affected by habitat (Pollard & Greatorex‐Davies, 1997; Altermatt, 2012) and microclimate (Wallisdevries & Van Swaay, 2006). The fact that we find a consistently stronger phenological response per 1 °C change over time compared with space (Fig. 2) suggests that compensation with respect to temperature is not the sole influence on local phenology. We would, however, expect such a pattern if local butterfly populations can function optimally within a range of temperatures experienced at each site.

The difference between spatial and temporal relationships found here might also arise if additional phenological cues elicit a plastic phenological response spatially but not temporally, for example photoperiod. Although photoperiod has a key role for insect development and phenology in seasonal environments (Nylin & Gotthard, 1998), it is not likely to explain the patterns we observe here. There are few examples of a photoperiod cue operating on the timing of butterfly emergence, and this cue appears to be more important for determining when insects enter diapause (Bradshaw & Holzapfel, 2007). Moreover, we find strong trends in emergence dates within populations over time, despite photoperiod being fixed at locations. Evidence for local adaptation is found for species with both relatively narrow and large latitudinal (and climatic) ranges in the UK (Table S2).

One explanation for the patterns in phenology–temperature patterns in butterflies is that countergradient local adaptation, whereby development is faster in colder areas (Conover & Schultz, 1995), may be prevalent in Lepidoptera. The adaptive explanation for such a countergradient in development rates is a trade‐off between a cost to the butterfly of emerging too early – perhaps in the form of exposure to late frosts for spring‐flying species – vs. the advantage of emerging early to maximise the growth and reproduction achieved during the summer months. Fitness costs of high growth rates (Conover & Present, 1990), such as increased exposure to predators, increase risk of desiccation, etc., may also lead to delays in development in warmer locations. These trade‐offs may give rise to geographic variation in the optimum average emergence date. Countergradient local adaptation may also be driven by host plants that themselves show a countergradient trend. However, in general trophic generalism makes it unlikely that patterns in butterfly phenology are driven by host‐plant availability, even though countergradients have been reported in plants (Eckhart *et al*., 2004). For the butterfly species analysed here, host availability *per se* is not likely to be limiting (Quinn *et al*., 1998) and, where investigated, butterfly phenology appears to be better predicted by temperature than by flowering times of host plants (Phillimore *et al*., 2012). Host‐plant quality is a key factor in the persistence of butterfly populations, however, and can vary with abiotic factors such as altitude, geology and climate. As such, variability in host‐plant use may be a mechanism causing spatial patterns in butterfly emergence dates. Some butterfly species are known to exploit differing food plants across their geographic range, and this can change through climatic conditions (Pateman *et al*., 2012; Bridle *et al*., 2014).

Our finding of substantial phylogenetic signal for the temporal slope, which we take to be a measure of phenological plasticity, is consistent with earlier work on the phylogenetic signal of phenological responses of plants to temperature in Thoreau's woods (Willis *et al*., 2008). Both results reveal a role for phylogenetic climatic niche conservatism. An implication of our finding of high phylogenetic signal in phenological plasticity is that we may be able to predict the phenological plasticity of species outside this study, provided that they are closely related to species we included here (Davis *et al*., 2010). The slope difference (degree of local adaptation) in comparison was found not to be phylogenetically heritable – although the credible interval was broad – suggesting that close relatives are not more likely to become locally adapted in either a countergradient or co‐gradient way.

Understanding these gradients and how they maintain population responses, including flight dates and population stability (Thomas *et al*., 1994; Oliver *et al*., 2010), are key to our ability to predict the impacts of climate change (Pau *et al*., 2011). If local adaptation to temperature occurs widely, as suggested here, this has implications for the conservation of butterflies by introduction from one locality to aid range expansion (Hoegh‐Guldberg *et al*., 2008) or where extinction has occurred. Butterflies moved from a cooler to a warmer locality may emerge too early in the season to interact with local resources, as occurred when *Maculinea arion* was introduced from Sweden to UK sites where mean temperatures were >2 °C cooler than the source (D. J. Simcox pers comm.). More importantly, a locally adapted butterfly may be unable to cope with predicted rapid climate warming, even if that increase remains well within the climate envelope of the species as a whole (Pelini *et al*., 2009; Van Dyck *et al*., 2015). Strong directional selection may be expected under such scenarios, and we recommend field studies to confirm this prediction.

Niche (bioclimate) models are a primary tool for identifying the risks of climate change and informing future conservation policy for biodiversity, and specifically butterflies (Settele *et al*., 2008). Such models assume constant relationships between occurrence and environmental conditions across a species' entire range. The evidence for widespread local adaptation reported here, combined with low dispersal ability of many species, suggests that the vulnerability of UK butterflies to projected warming may be critically underestimated. Common garden experiments and reciprocal transplants (Hereford, 2009) are a priority to confirm the extent of local adaptation suggested by correlative models.

A fuller assessment of the extent of local adaptations within populations and greater understanding of the underlying mechanisms are essential for more accurate projections of the impacts of climate warming on biodiversity and the ecosystem services it supports (Visser, 2008).

## Author contributions

DR designed the study; MB, TB, BB CH, RD collected and processed data; TO and AP designed and performed the analysis; DR, TO, AP and JT wrote the manuscript.

## Supporting information


**Data S1.** Developing a phylogeny of British butterflies (technical details).
**Table S1.** Species' t‐values and co‐efficients from regressions of mean flight date and three‐monthly mean temperatures.
**Table S2.** Temperature range (within‐ and between‐ populations) of data analysed for each species.
**Table S3.** Results from phylogenetic MCMCglmm analysis.
**Figure S1.** Locations of the 1622 United Kingdom Butterfly Monitoring Scheme transects used for the analysis.
**Figure S2.** Expected shift in mean flight date for a 1 °C increase in mean temperature in both the spatial (*x*‐axis) and temporal dimension (*y*‐axis) for the 30 species analysed.
**Figure S3.** Expected shift in mean flight date for a 1 °C increase in mean temperature in both the spatial (*x*‐axis) and temporal dimension (*y*‐axis) for the 30 species analysed.
**Figure S4.** Plots of flight date on temperature for each species.
**Figure S5.** The maximum clade credibility mtDNA COI gene tree for British butterflies obtained using Beast ^[1]^ with branch lengths proportional to time.
