# Supplementary material for: Similarities in butterfly emergence dates among populations suggest local adaptation to climate
Source: Glob Chang Biol. 2015 Jun 17;21(9):3313–22. doi: 10.1111/gcb.12920 (PMC4744750; doi:10.1111/gcb.12920)
Supplement: Supplementary file 1 — Data S1. Developing a phylogeny of British butterflies (technical details). Table S1. Species' t‐values and co‐efficients from regressions of mean flight date and three‐monthly mean temperatures. Table S2. Temperature range (within‐ and between‐ populations) of data analysed for each species. Table S3. Results from phylogenetic MCMCglmm analysis. Figure S1. Locations of the 1622 United Kingdom Butterfly Monitoring Scheme transects used for the analysis. Figure S2. Expected shift in mean flight date for a 1 °C increase in mean temperature in both the spatial (x‐axis) and temporal dimension (y‐axis) for the 30 species analysed. Figure S3. Expected shift in mean flight date for a 1 °C increase in mean temperature in both the spatial (x‐axis) and temporal dimension (y‐axis) for the 30 species analysed. Figure S4. Plots of flight date on temperature for each species. Figure S5. The maximum clade credibility mtDNA COI gene tree for British butterflies obtained using Beast [1] with branch lengths proportional to time. [file GCB-21-3313-s001.docx]

**Supplementary material**

*Developing a phylogeny of British butterflies (technical details)*

Phylogeny reconstruction used a relaxed-clock Bayesian approach ([Drummond *et al.*, 2006a](#_ENREF_9)) implemented in Beast v1.5.4 ([Drummond and Rambaut, 2007](#_ENREF_11)). We constrained several sets of species that correspond to well-supported clades in two recent higher level phylogenies of butterflies that used multiple genes and morphological data ([Mutanen *et al.*, 2010](#_ENREF_20); [Wahlberg *et al.*, 2009](#_ENREF_50)) (constrained nodes are indicated on phylogeny). We used the SRD06 codon model that allows the substitution rates to differ between codon position 3 versus positions 1 and 2 ([Shapiro *et al.*, 2006](#_ENREF_42)). We used a pure birth tree prior and random starting tree and a HKY + Γ substitution model. We assumed that substitution rate heterogeneity was lognormally distributed and uncorrelated, with the mean substitution rate set at 1. We conducted four runs of 50 million generations, sampling from the posterior distribution every 5000 generations. To assess mixing, that all independent runs were sampling from the posterior distribution and that the estimated sample sizes for all parameters were adequate (>200) we used Tracer v1.5 (http://tree.bio.ed.ac.uk/software/tracer/) after removing the first 5million generations as burnin. We built a maximum clade credibility tree from 36,000 samples drawn from the posterior distribution (Figure S5). For use in comparative analyses we obtained a posterior sample of 1000 trees (Appendix file S1) from which we selected only those species for which phenology had been assessed.

Table S1. Species’ t-values and co-efficients from regressions of mean flight date and three-monthly mean temperatures. The month with the largest value for each species is given in bold.

a) **t-values** from regressions of mean flight date and three-monthly mean temperatures

| Species | January | February | March | April | May | June | July | August | September | October | November | December |
| --- | --- | --- | --- | --- | --- | --- | --- | --- | --- | --- | --- | --- |
| *Thymelicus sylvestris* | -2.1 | -6.3 | -19.6 | -24.0 | **-37.4** | -32.1 | -27.1 | -21.7 | -21.1 | -23.4 | -12.8 | -12.3 |
| *Hesperia comma* | -1.7 | -1.8 | -1.6 | -1.1 | -3.5 | -6.1 | **-7.4** | -5.5 | -2.4 | -1.5 | 0.0 | -2.1 |
| *Ochlodes sylanus* | -2.0 | -9.0 | -21.9 | -21.2 | **-27.6** | -12.6 | -4.5 | -0.8 | -3.3 | -9.8 | -5.1 | -10.0 |
| *Erynnis tages* | -5.5 | -14.7 | **-25.1** | -24.0 | -20.0 | -6.2 | -4.0 | -5.9 | -9.0 | -9.4 | -7.5 | -9.4 |
| *Pyrgus malvae* | -1.2 | -9.8 | **-20.1** | -18.6 | -15.5 | -1.4 | 0.1 | -1.2 | -5.0 | -8.0 | -7.5 | -8.0 |
| *Anthocharis cardamines* | -18.1 | -39.4 | **-63.5** | -63.0 | -53.5 | -23.0 | -15.5 | -17.0 | -24.1 | -31.9 | -29.7 | -31.6 |
| *Callophrys rubi* | -0.2 | -7.4 | **-12.9** | -11.1 | -8.0 | 2.1 | 5.0 | 2.8 | -0.2 | -2.9 | -2.5 | -4.2 |
| *Polyommatus coridon* | -3.6 | -5.3 | -7.4 | -7.9 | -10.0 | **-12.0** | -11.4 | -10.0 | -6.7 | -6.2 | -4.3 | -5.9 |
| *Limenitis camilla* | -5.8 | -10.6 | -17.4 | -20.5 | **-26.1** | -15.6 | -7.3 | -6.3 | -7.5 | -11.4 | -7.9 | -10.8 |
| *Boloria selene* | -12.6 | -15.2 | -19.3 | -21.1 | **-23.0** | -18.8 | -16.4 | -15.4 | -16.1 | -15.1 | -12.2 | -12.3 |
| *Boloria euphrosyne* | -13.5 | -18.5 | **-24.4** | -22.5 | -19.5 | -12.0 | -10.4 | -11.2 | -13.3 | -14.6 | -13.5 | -15.4 |
| *Argynnis adippe* | -2.1 | -3.3 | -6.1 | -7.1 | **-9.7** | -6.4 | -5.0 | -4.0 | -3.7 | -3.8 | -1.3 | -2.5 |
| *Argynnis aglaja* | -4.2 | -5.5 | -9.6 | -9.9 | **-12.4** | -10.2 | -9.3 | -8.1 | -8.4 | -8.6 | -6.4 | -6.4 |
| *Euphydryas aurinia* | -0.9 | -5.9 | -12.5 | **-13.7** | -12.2 | -4.5 | -2.3 | -3.0 | -5.0 | -5.9 | -3.7 | -4.1 |
| *Argynnis paphia* | 0.4 | -4.4 | -13.7 | -16.0 | **-21.1** | -16.8 | -14.3 | -13.7 | -13.9 | -13.8 | -9.0 | -8.1 |
| *Melanargia galathea* | 2.3 | -5.3 | -22.3 | -27.2 | **-38.1** | -24.0 | -16.5 | -12.1 | -12.0 | -13.9 | -5.2 | -5.9 |
| *Hipparchia semele* | 1.6 | 1.0 | -1.0 | -0.4 | **-2.0** | -0.9 | 0.1 | 1.2 | 2.1 | 1.1 | 1.9 | 0.5 |
| *Pyronia tithonus* | -7.5 | -5.9 | -12.1 | -19.6 | -38.9 | **-56.3** | -51.0 | -43.5 | -30.4 | -23.6 | -9.4 | -9.6 |
| *Maniola jurtina* | 0.1 | -4.6 | -16.1 | -19.7 | **-29.7** | -28.1 | -23.9 | -20.1 | -17.4 | -17.9 | -8.2 | -6.7 |
| *Aphantopus hyperantus* | -3.1 | -12.4 | -28.4 | -32.8 | **-41.5** | -25.7 | -16.2 | -14.4 | -15.4 | -21.1 | -13.3 | -15.1 |
| *Gonepteryx rhamni* | -7.4 | -9.8 | -16.5 | -18.3 | -27.0 | -31.8 | **-34.1** | -32.1 | -27.4 | -21.2 | -10.3 | -10.7 |
| *Aglais io* | -14.8 | -20.5 | -36.6 | -44.8 | **-62.6** | -59.9 | -53.6 | -49.3 | -46.1 | -41.1 | -24.7 | -23.1 |
| *Pieris rapae* | -11.3 | -25.8 | -44.3 | **-46.3** | -42.9 | -20.0 | -14.9 | -15.9 | -20.4 | -24.4 | -19.2 | -19.8 |
| *Pieris napi* | -19.2 | -35.8 | -57.6 | **-60.9** | -55.2 | -31.1 | -26.1 | -27.2 | -30.8 | -34.4 | -29.0 | -29.5 |
| *Pieris brassicae* | -10.9 | -24.8 | -41.2 | **-45.1** | -41.2 | -19.1 | -12.8 | -13.2 | -17.1 | -21.5 | -17.1 | -17.7 |
| *Lycaena phlaeas* | -13.0 | -22.5 | **-32.5** | -31.0 | -24.9 | -12.4 | -8.7 | -10.8 | -15.4 | -17.8 | -16.7 | -18.3 |
| *Cupido minimus* | -5.5 | -10.7 | **-14.4** | -13.8 | -11.6 | -2.8 | 0.5 | -0.5 | -4.5 | -6.4 | -6.0 | -7.2 |
| *Aricia agestis* | -5.6 | -15.2 | **-25.3** | -23.4 | -19.3 | -3.0 | 0.8 | -2.0 | -8.9 | -12.4 | -11.1 | -11.9 |
| *Polyommatus icarus* | -16.8 | -29.7 | -46.5 | **-49.2** | -48.0 | -27.3 | -20.9 | -21.4 | -28.2 | -29.5 | -24.4 | -24.0 |
| *Celastrina argiolus* | -8.6 | -21.0 | **-33.5** | -29.1 | -21.4 | -1.1 | 3.5 | 0.7 | -6.9 | -14.7 | -17.6 | -20.0 |
| *Lasiommata megera* | -20.8 | -27.0 | **-32.7** | -29.5 | -22.1 | -10.1 | -6.1 | -7.7 | -11.7 | -12.5 | -11.7 | -15.5 |

b) **co-efficients** from regressions of mean flight date and three-monthly mean temperatures

| Species | January | February | March | April | May | June | July | August | September | October | November | December |
| --- | --- | --- | --- | --- | --- | --- | --- | --- | --- | --- | --- | --- |
| *Thymelicus sylvestris* | -0.23 | -0.73 | -2.46 | -3.48 | **-5.23** | -4.68 | -3.73 | -2.99 | -2.91 | -2.96 | -1.51 | -1.33 |
| *Hesperia comma* | -0.53 | -0.59 | -0.59 | -0.48 | -1.63 | -2.82 | **-3.05** | -2.16 | -0.95 | -0.54 | 0.00 | -0.68 |
| *Ochlodes sylanus* | -0.20 | -0.95 | -2.50 | -2.83 | **-3.66** | -1.74 | -0.58 | -0.10 | -0.42 | -1.13 | -0.54 | -0.98 |
| *Erynnis tages* | -0.95 | -2.65 | -4.72 | **-5.39** | -4.82 | -1.65 | -0.96 | -1.39 | -1.99 | -1.87 | -1.36 | -1.60 |
| *Pyrgus malvae* | -0.31 | -2.67 | -5.51 | **-6.31** | -5.71 | -0.57 | 0.05 | -0.43 | -1.71 | -2.40 | -2.09 | -2.03 |
| *Anthocharis cardamines* | -2.00 | -4.27 | -6.56 | **-7.41** | -6.72 | -3.33 | -2.09 | -2.27 | -3.17 | -3.85 | -3.41 | -3.37 |
| *Callophrys rubi* | -0.05 | -1.71 | **-3.14** | -3.07 | -2.31 | 0.63 | 1.34 | 0.73 | -0.04 | -0.70 | -0.58 | -0.92 |
| *Polyommatus coridon* | -0.67 | -1.06 | -1.66 | -2.13 | -2.81 | **-3.44** | -2.97 | -2.49 | -1.64 | -1.34 | -0.86 | -1.08 |
| *Limenitis camilla* | -1.19 | -2.20 | -3.75 | -5.25 | **-6.79** | -4.80 | -2.21 | -1.78 | -2.05 | -2.70 | -1.71 | -2.07 |
| *Boloria selene* | -2.96 | -3.64 | -4.83 | -5.67 | **-6.24** | -5.22 | -4.33 | -4.00 | -4.16 | -3.90 | -3.00 | -2.87 |
| *Boloria euphrosyne* | -4.11 | -5.04 | -6.49 | **-6.85** | -6.60 | -4.55 | -3.78 | -3.92 | -4.50 | -4.86 | -4.40 | -4.65 |
| *Argynnis adippe* | -0.82 | -1.30 | -2.62 | -3.71 | **-5.52** | -3.79 | -2.68 | -2.15 | -2.01 | -1.91 | -0.59 | -1.02 |
| *Argynnis aglaja* | -0.80 | -1.10 | -2.00 | -2.27 | **-2.80** | -2.28 | -1.96 | -1.68 | -1.75 | -1.72 | -1.22 | -1.15 |
| *Euphydryas aurinia* | -0.34 | -2.36 | -4.96 | **-6.55** | -6.35 | -2.43 | -1.08 | -1.39 | -2.44 | -2.93 | -1.75 | -1.60 |
| *Argynnis paphia* | 0.07 | -0.94 | -3.10 | -4.31 | **-5.68** | -4.76 | -3.75 | -3.48 | -3.42 | -2.96 | -1.73 | -1.44 |
| *Melanargia galathea* | 0.24 | -0.60 | -2.68 | -3.85 | **-5.22** | -3.66 | -2.36 | -1.69 | -1.65 | -1.65 | -0.57 | -0.59 |
| *Hipparchia semele* | 0.43 | 0.28 | -0.33 | -0.15 | **-0.66** | -0.29 | 0.02 | 0.37 | 0.64 | 0.33 | 0.53 | 0.12 |
| *Pyronia tithonus* | -0.46 | -0.39 | -0.89 | -1.70 | -3.20 | -4.39 | **-3.77** | -3.26 | -2.40 | -1.69 | -0.63 | -0.58 |
| *Maniola jurtina* | 0.00 | -0.38 | -1.41 | -1.90 | **-2.75** | -2.56 | -2.06 | -1.73 | -1.53 | -1.52 | -0.66 | -0.51 |
| *Aphantopus hyperantus* | -0.22 | -0.94 | -2.27 | -2.97 | **-3.60** | -2.35 | -1.40 | -1.24 | -1.32 | -1.65 | -0.97 | -1.03 |
| *Gonepteryx rhamni* | -1.11 | -1.53 | -2.80 | -3.69 | -5.42 | **-6.51** | -6.30 | -5.86 | -5.09 | -3.70 | -1.70 | -1.59 |
| *Aglais io* | -1.48 | -2.15 | -3.99 | -5.38 | **-6.97** | -6.78 | -5.78 | -5.36 | -5.16 | -4.46 | -2.64 | -2.30 |
| *Pieris rapae* | -1.54 | -3.55 | -6.22 | **-7.47** | -7.09 | -3.63 | -2.52 | -2.65 | -3.40 | -3.76 | -2.78 | -2.67 |
| *Pieris napi* | -1.91 | -3.50 | -5.50 | **-6.36** | -5.90 | -3.65 | -2.90 | -2.97 | -3.38 | -3.63 | -2.96 | -2.85 |
| *Pieris brassicae* | -1.40 | -3.23 | -5.52 | **-6.91** | -6.43 | -3.23 | -2.03 | -2.06 | -2.68 | -3.11 | -2.33 | -2.26 |
| *Lycaena phlaeas* | -2.22 | -3.99 | -5.88 | **-6.31** | -5.28 | -2.88 | -1.93 | -2.39 | -3.33 | -3.52 | -3.02 | -3.06 |
| *Cupido minimus* | -1.49 | -2.96 | -4.23 | **-4.87** | -4.37 | -1.13 | 0.17 | -0.18 | -1.56 | -1.97 | -1.66 | -1.82 |
| *Aricia agestis* | -1.29 | -3.69 | -5.94 | **-6.76** | -6.19 | -1.18 | 0.28 | -0.69 | -2.85 | -3.27 | -2.66 | -2.60 |
| *Polyommatus icarus* | -1.98 | -3.64 | -5.84 | **-7.06** | -6.93 | -4.28 | -3.06 | -3.09 | -4.00 | -3.86 | -2.93 | -2.69 |
| *Celastrina argiolus* | -1.46 | -3.76 | -6.05 | **-6.52** | -4.96 | -0.29 | 0.85 | 0.18 | -1.63 | -2.96 | -3.22 | -3.35 |
| *Lasiommata megera* | -4.14 | -5.14 | -6.84 | **-7.69** | -6.57 | -3.24 | -1.83 | -2.28 | -3.31 | -3.32 | -2.78 | -3.37 |

Table S2. Temperature range (within- and between- populations) of data analysed for each species. The full and inter-quartile range is included for each species.

| Species | Between-populations | | Within-populations | |
| --- | --- | --- | --- | --- |
|  | Full range | Inter-quartile range | Full range | Inter-quartile range |
| *Thymelicus sylvestris* | 5.97 | 1.01 | 3.01 | 1.06 |
| *Hesperia comma* | 2.29 | 0.61 | 3.83 | 1.27 |
| *Ochlodes sylvanus* | 6.923 | 0.97 | 3.203 | 1.17 |
| *Erynnis tages* | 6.833 | 0.868 | 5.188 | 1.313 |
| *Pyrgus malvae* | 6.343 | 0.882 | 5.463 | 1.159 |
| *Anthocharis cardamines* | 8.458 | 0.95 | 5.287 | 1.229 |
| *Callophrys rubi* | 10.5037 | 0.947 | 5.415 | 1.262 |
| *Polyommatus coridon* | 4.14 | 0.77 | 3.46 | 1.26 |
| *Limenitis camilla* | 3.72 | 0.76 | 3.594 | 1.27 |
| *Boloria selene* | 7.333 | 1.31 | 3.348 | 0.92 |
| *Boloria euphrosyne* | 8.195 | 1.277 | 5.562 | 1.511 |
| *Argynnis adippe* | 3.544 | 0.62 | 3.022 | 0.94 |
| *Argynnis aglaja* | 7.026 | 1.27 | 2.809 | 1.34 |
| *Euphydryas aurinia* | 3.827 | 0.744 | 3.217 | 0.931 |
| *Argynnis paphia* | 4.763 | 0.91 | 3.09 | 1.14 |
| *Melanargia galathea* | 5.063 | 0.87 | 3.22 | 1.2 |
| *Hipparchia semele* | 6.713 | 1.33 | 3.86 | 1.1 |
| *Pyronia tithonus* | 5.53 | 0.88 | 3.3 | 1.09 |
| *Maniola jurtina* | 8.504 | 1.14 | 3.272 | 1.23 |
| *Aphantopus hyperantus* | 8.44 | 1.01 | 2.975 | 1.01 |
| *Gonepteryx rhamni* | 6.47 | 0.85 | 3.19 | 1.15 |
| *Aglais io* | 7.977 | 1.1 | 3.12 | 1.13 |
| *Pieris rapae* | 8.876 | 0.899 | 3.258 | 1.256 |
| *Pieris napi* | 8.923 | 0.927 | 3.405 | 1.392 |
| *Pieris brassicae* | 8.661 | 0.876 | 3.321 | 1.334 |
| *Lycaena phlaeas* | 9.0096 | 1.18 | 5.178 | 1.304 |
| *Cupido minimus* | 5.313 | 0.853 | 5.463 | 1.315 |
| *Aricia agestis* | 5.08 | 1.125 | 5.717 | 1.324 |
| *Polyommatus icarus* | 6.797 | 0.925 | 3.21 | 1.314 |
| *Celastrina argiolus* | 5.553 | 0.952 | 5.307 | 1.279 |
| *Lasiommata megera* | 6.804 | 1.146 | 5.104 | 1.325 |

Table S3, Results from phylogenetic MCMCglmm analysis

|  | **Within population slope** | | | **Slope difference** | | |
| --- | --- | --- | --- | --- | --- | --- |
|  | **Slope** | **95% interval** | | **Slope** | **95% interval** | |
| **Variable** |  | **Lower** | **Upper** |  | **Lower** | **Upper** |
| **Intercept** | **-5.61** | **-9.06** | **-2.30** | 5.46 | -4.21 | 14.86 |
| Voltinism | -0.15 | -1.23 | 0.83 | -1.58 | -4.25 | 1.41 |
| Larval duration | 0.001 | -0.001 | 0.001 | -0.01 | -0.03 | 0.02 |
| Hibernation stage | -0.23 | -1.07 | 0.58 | 0.84 | -1.55 | 3.22 |
| Mobility | -0.18 | -0.46 | 0.11 | -0.36 | -1.14 | 0.42 |
| mean flight date | 0.01 | -0.01 | 0.02 | -0.01 | -0.05 | 0.03 |

Figure S1. Locations of the 1622 United Kingdom Butterfly Monitoring Scheme transects used for the analysis.


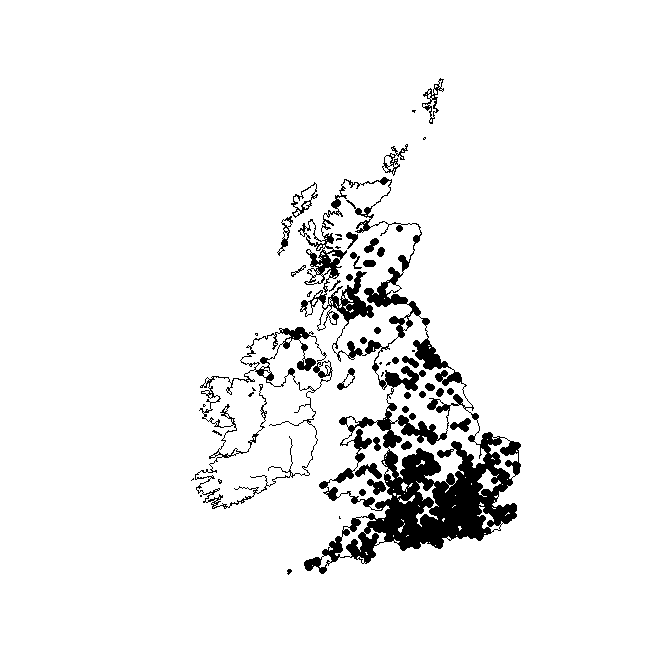


Figure S2. Expected shift in mean flight date for a 1ºC increase in mean temperature in both the spatial (x-axis) and temporal dimension (y-axis) for the 30 species analysed. The line of unity indicates the null hypothesis that temperature change over both space and time has the same effect on phenological shift.

**Using (a) coefficients or (b) t-values from regressions of mean flight date and three-monthly mean temperature.**

**a) b)**

**
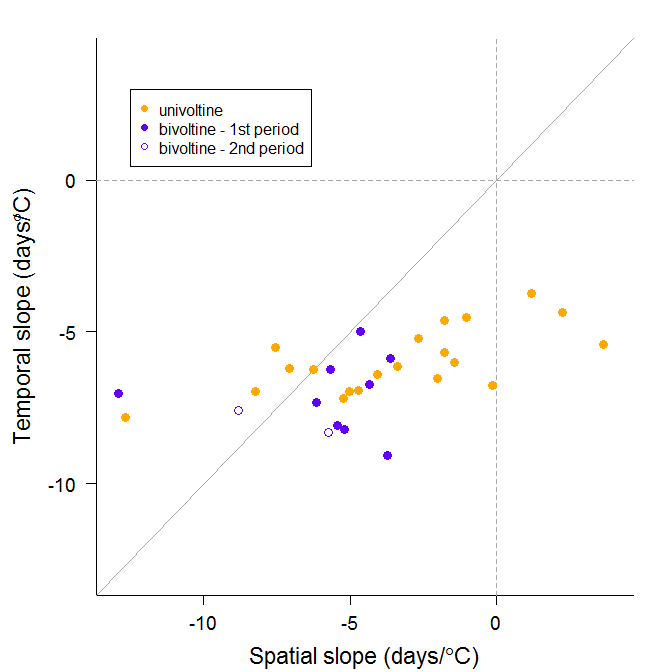
**

Figure S3. Expected shift in mean flight date for a 1ºC increase in mean temperature in both the spatial (x-axis) and temporal dimension (y-axis) for the 30 species analysed. The line of unity indicates the null hypothesis that temperature change over both space and time has the same effect on phenological shift.

**Using (a) site level (b) 50km grid square and (c) 100km grid square.**

**a) b) c)**


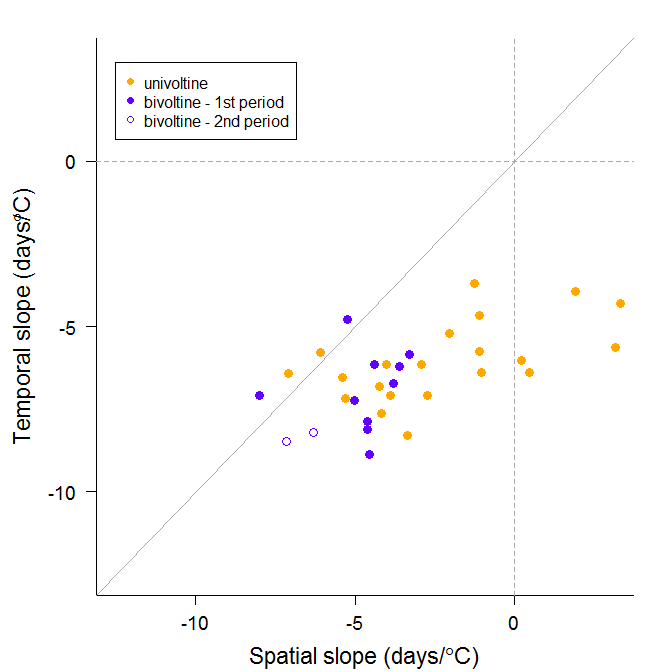
**
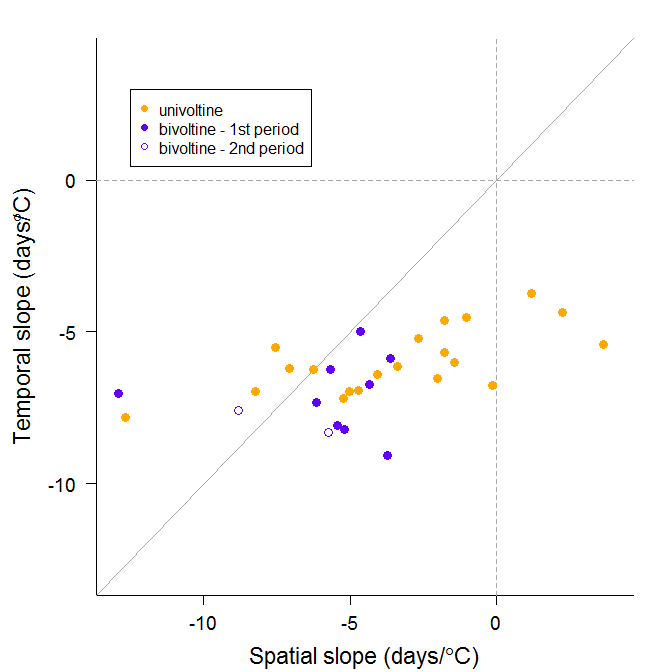
**
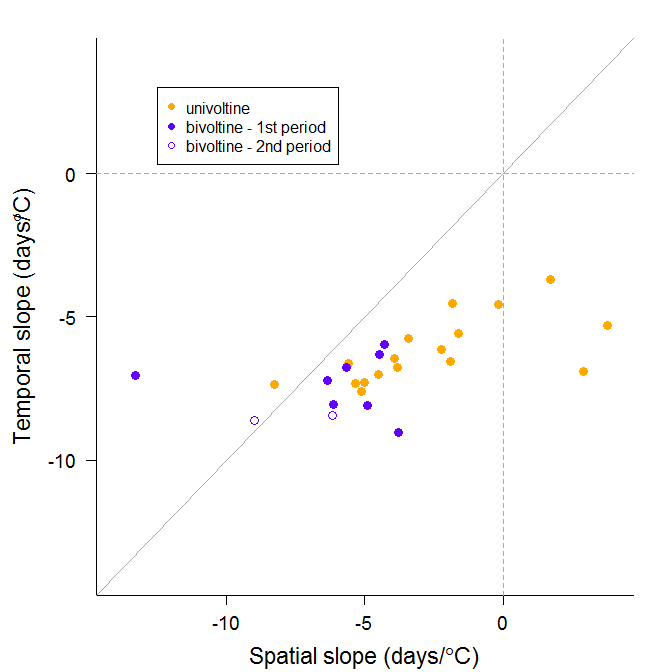


Figure S4, Plots of flight date on temperature for each species. A local regression was fitted for each 50km^2^ population. To ensure data quality, only sites with greater than five years of data were included in the analysis and only 50km^2^ populations with at least 30 datapoints. Species represented in each panel are as follows: a) *Aglais urticae,* b) *Aphantopus hyperantus,* c) *Argynnis aglaja ,* d) *Argynnis adippe, e) Boloria euphrosyne,* f) *Argynnis paphia, g*) *Boloria selene, h) Aricia agestis, i) Aricia artaxerxes, j) Callophyrs rubi, k) Celastrina argiolus, l) Cupido minimus, m) Erynnis tages, n) Hipparchia semele, o)* ) *Euphydryas aurinia, p) Gonepteryx rhamni, q) Hesperia comma, r) Limenitis camilla, s) Lycaena phlaeas, t) Polyommatus coridon, u) Maniola jurtina, v) Pyronia tithonus, w) Melanargia galathea, x) Aglais io, y) Ochlodes sylvanus, Lasiommata megera, az) Pieris brassicae, bz) Pieris napi, cz) Pieris rapae, dz) Polyommatus icarus, ez) Pyrgus malvae, fz) Thymelicus sylvestris.*


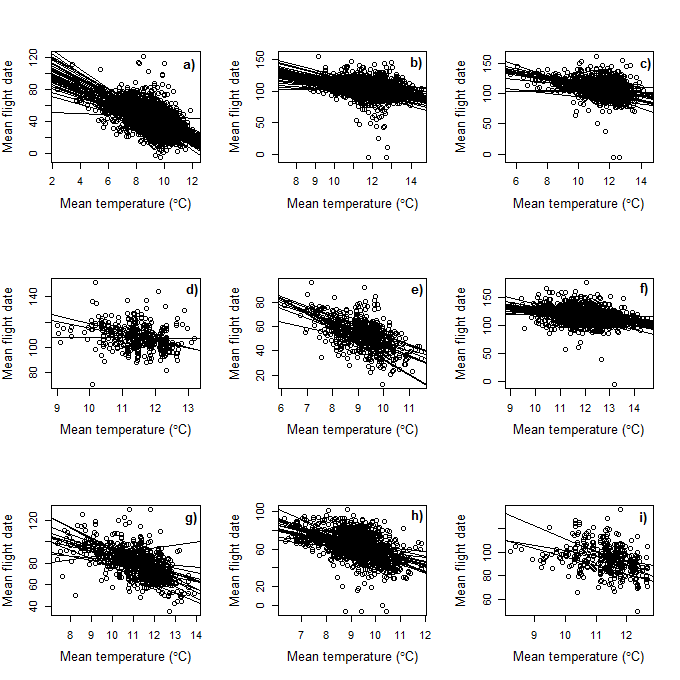


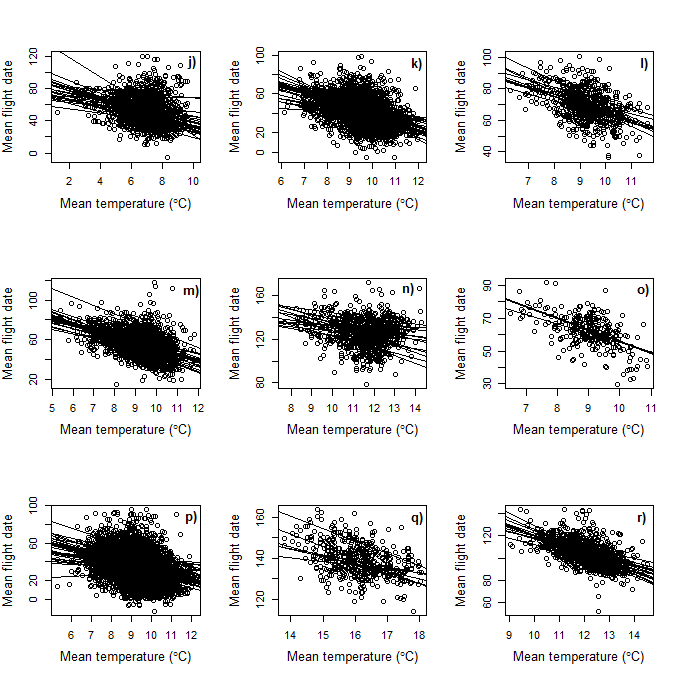


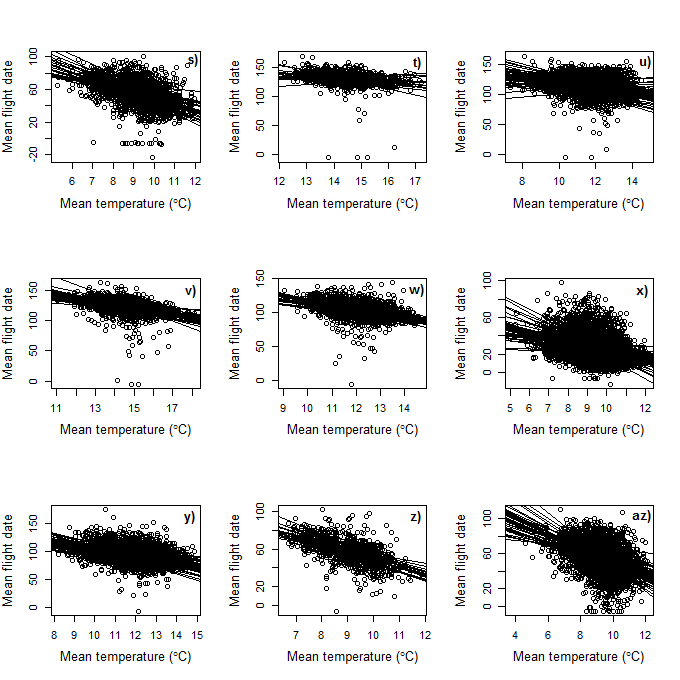


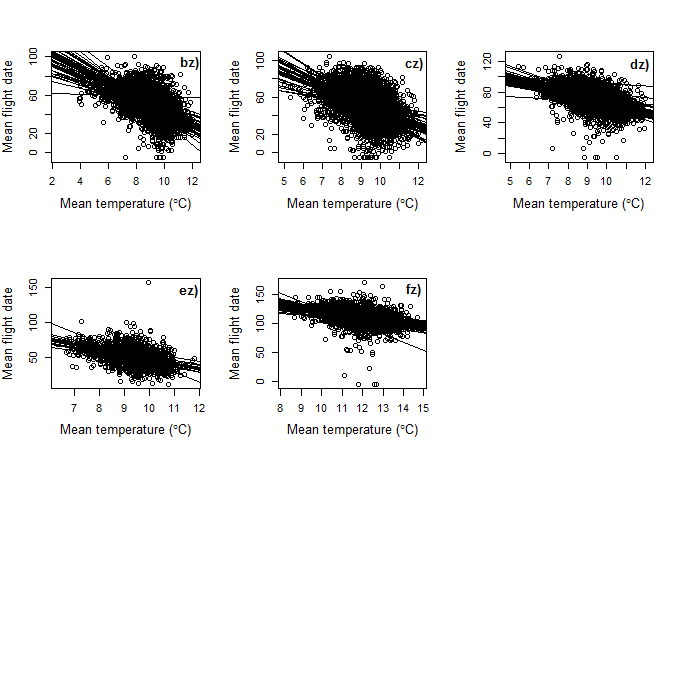


Figure S5. The maximum clade credibility mtDNA COI gene tree for British butterflies obtained using Beast ^[^[^1^](#_ENREF_1)^]^ with branch lengths proportional to time. Numbers that follow species names indicate British species that were substituted by non-British congeners, the substituted species are as follows; [1] *Erynnis tages*, [2] *Thymelicus acteon*, *T. lineola* and *T. sylvestris*; [3] *Ochlodes sylvanus*; [4] *Hesperia comma*; [5] *Satyrium pruni* and *S. w-album*. Values below nodes are Bayesian posterior probabilities. Letters at nodes indicate clades that were constrained to be monophyletic based on recent multi-locus higher-level Lepidoptera phylogenies (a,c,d,e,f ^[^[^2^](#_ENREF_2)^]^ and b ^[^[^3^](#_ENREF_3)^]^).

## References

1. Drummond, A.J. and A. Rambaut, *BEAST: Bayesian evolutionary analysis by sampling trees.* BMC Evolutionary Biology, 2007. **7**: p. 214.

2. Wahlberg, N., et al., *Nymphalid butterflies diversify following near demise at the Cretaceous/Tertiary boundary.* Proc. R. Soc. Lond. B, 2009. **276**: p. 4295-4302.

3. Mutanen, M., N. Wahlberg, and L. Kaila, *Comprehensive gene and taxon coverage elucidates radiation patterns in moths and butterflies.* Proc. R. Soc. Lond. B, 2010. **In Press**.
